# *TCF7L2* polymorphisms are associated with amygdalar volume in elderly individuals with Type 2 Diabetes

**DOI:** 10.1038/s41598-019-48899-3

**Published:** 2019-11-01

**Authors:** Ithamar Ganmore, Abigail Livny, Ramit Ravona-Springer, Itzik Cooper, Anna Alkelai, Shahar Shelly, Galia Tsarfaty, Anthony Heymann, Michal Schnaider Beeri, Lior Greenbaum

**Affiliations:** 1 Department of Neurology, Sheba Medical Center, Tel Hashomer, Ramat Gan, Israel; 2 The Joseph Sagol Neuroscience Center, Sheba Medical Center, Tel Hashomer, Ramat Gan, Israel; 3 Department of Diagnostic Imaging, Sheba Medical Center, Tel Hashomer, Ramat Gan, Israel; 4 Memory clinic, Sheba Medical Center, Tel Hashomer, Ramat Gan, Israel; 5 Sackler Faculty of Medicine, Tel Aviv University, Tel Aviv, Israel; 6 Institute for Genomic Medicine, Columbia University, New York, NY USA; 7 Department of Neurology, Mayo Clinic, Rochester, Minnesota USA; 8 Maccabi Healthcare Services, Tel Aviv, Israel; 9 Department of Psychiatry, Icahn School of Medicine at Mount Sinai, New York, NY USA; 10 The Danek Gertner Institute of Human Genetics, Sheba Medical Center, Tel Hashomer, Ramat Gan, Israel

**Keywords:** Type 2 diabetes, Genetics research

## Abstract

The association between several Single Nucleotide Polymorphisms (SNPs) within the transcription factor 7-like 2 (*TCF7L2)* gene and Type 2 Diabetes (T2D) as well as additional T2D-related traits is well established. Since alteration in total and regional brain volumes are consistent findings among T2D individuals, we studied the association of four T2D susceptibility SNPS within *TCF7L2* (rs7901695, rs7903146, rs11196205, and rs12255372) with volumes of white matter hyperintensities (WMH), gray matter, and regional volumes of amygdala and hippocampus obtained from structural MRI among 191 T2D elderly Jewish individuals. Under recessive genetic model (controlling for age, sex and intracranial volume), we found that for all four SNPs, carriers of two copies of the T2D risk allele (homozygous genotype) had significantly smaller amygdalar volume: rs7901695- CC genotype vs. CT + TT genotypes, p = 0.002; rs7903146-TT vs. TC + CC, p = 0.003; rs11196205- CC vs. CG + GG, p = 0.0003; and rs12255372- TT vs. TG + GG, p = 0.003. Adjusting also for T2D-related covariates, body mass index (BMI), and ancestry did not change the results substantively (rs7901695, p = 0.003; rs7903146, p = 0.005; rs11196205, p = 0.001; and rs12255372, p = 0.005). Conditional analysis demonstrated that only rs11196205 was independently associated with amygdalar volume at a significant level. Separate analysis of left and right amygdala revealed stronger results for left amygdalar volume. Taken together, we report association of *TCF7L2* SNPs with amygdalar volume among T2D elderly Jewish patients. Further studies in other populations are required to support these findings and reach more definitive conclusions.

## Introduction

Type 2 diabetes (T2D) is a multifactorial disease, with a complex polygenic architecture^1–3^. The association of the transcription factor 7-like 2 (*TCF7L2*) gene (chromosome 10q25.2-q25.3) with T2D is one of the most reproducible and robust finding in T2D genetics, as supported by Genome-wide association studies (GWAS), multiple replication studies and meta-analyses^4,5^. Several single nucleotide polymorphisms (SNPs) within *TCF7L2* were independently associated with T2D susceptibility and related traits (e.g. insulin secretion and blood glucose levels)^4–8^.

The protein encoded by *TCF7L2* gene is a transcription factor, involved in the Wnt/beta-catenin signaling pathway, which plays a role in cell proliferation and differentiation^9,10^. It is related to beta-cells and other pancreatic cells functions^11–13^, as well as to the development and function of adipose tissue^14^. In addition, *TCF7L2* is expressed in multiple brain regions and characterized by existence of several alternative splicing variants in different species^15–18^. In humans, a unique splice variant was found in the brain, pancreatic islets and gut and therefore named the “neuroendocrine form“^19^. SNPs in *TCF7L2* have been associated with psychiatric disorders, such as schizophrenia and bipolar disorder^20–22^.

Individuals with T2D have higher risk for cognitive dysfunction and dementia than those without T2D^23^. The most consistent findings in neuroimaging of T2D patients are higher number of infarcts and white matter lesions, as well as general cerebral and hippocampal atrophy^24–26^. Few studies have found regional atrophy in different brain structures^27–29^, but these results were not consistent. Greater atrophy of the amygdala and hippocampus had been associated not only with T2D^30^, but also to high plasma glucose levels within the normal range^31^. However, the underlying etiology of brain volume differences in T2D is still unknown. At the genetic level, previous studies in the Israel Diabetes and Cognitive Decline (IDCD) study found that apolipoprotein ε4 (*APOE4*) genotype^32^ and haptoglobin (Hp) 1–1 genotype^33^, affect the relationship between neuroimaging phenotypes (White matter hyperintensities [WMHs] and Hippocampal volume, respectively) and glycemic control among T2D patients.

We examined the association of four *TCF7L2* SNPs (rs7901695, rs7903146, rs11196205, and rs12255372) with WMH, gray matter, and regional volumes of the amygdala and hippocampus obtained from structural brain magnetic resonance imaging (MRI) in Jewish T2D elderly patients. These SNPs are reported in the literature as robustly associated with T2D^4,5^. We hypothesized that *TCF7L2* risk alleles for T2D would be associated with smaller regional brain volume and larger WMH volume among T2D patients.

## Results

### Demographic and medical characteristics

The final analysis included 191 T2D individuals, all of them were IDCD participants. The demographic and clinical description of the sample is detailed in Table 1. All participants were both genotyped for the *TCF7L2* SNPs (rs7901695, rs7903146, rs11196205, and rs12255372; Table 2) and had brain MRI scans. Genotyping success rate per SNP was 97.38–100%

**Table 1:** Demographic and clinical description of the analyzed sample (N = 191).

Characteristics	Values
Sex (males/females, N [%])	118/73 (61.8%/38.2%)
Age at IDCD study baseline (Mean ± SD, years)	70.79 ± 4.22
Follow up in the registry (Mean ± SD, years)	9.33 ± 4.58
HbA1c (Mean ± SD, %)	6.66 ± 0.76
BMI (Mean ± SD, kg/m^2^)	28.37 ± 4.32
Systolic blood pressure (Mean ± SD, mmHg)	133.05 ± 9.32
Diastolic blood pressure (Mean ± SD, mmHg)	76.77 ± 4.94
T2D medications consumption (yes/no, N [%])	164/27 (85.9%/14.1%)
Ashkenazi origin (yes/no, N [%])	127/64 (66.5%/33.5%)

Abbreviations: N- number of subjects; SD- standard deviation; BMI- body mass index; HbA1c- hemoglobin A1c; T2D- type 2 diabetes; IDCD- the Israel Diabetes and Cognitive Decline.

**Table 2:** *TCF7L2* SNPs included in the analysis.

SNP	Position	Location	Minor, Major alleles	MAF	T2D risk allele
rs7901695	114754088	Intron 4	C, T	0.403	C
rs7903146	114758349	Intron 4	T, C	0.388	T
rs11196205	114807047	Intron 5	G, C	0.487	C
rs12255372	114808902	Intron 5	T, G	0.382	T

SNPs’ position refers to GRCh37.p13.

Abbreviations: SNP- single nucleotide polymorphism; MAF-minor allele frequency; T2D- type 2 diabetes.

### Association of *TCF7L2* SNPs with the volume of different brain regions

We found that two SNPs, rs7903146 and rs7901695 were highly correlated to each other (r^2^ = 0.94) and can be viewed essentially as a single signal (Table 3). From the analyzed SNPs, only rs7903146 showed slight deviation form Hardy-Weinberg equilibrium (p = 0.045), but nevertheless we included it in the analysis.

**Table 3:** Correlation (r^2^) and D’ values between analyzed *TCF7L2* SNPs.

SNP		r^2^			
		rs7901695	rs7903146	rs11196205	rs12255372
**D'**	**rs7901695**		0.94	0.62	0.81
	**rs7903146**	1		0.58	0.76
	**rs11196205**	0.97	0.97		0.61
	**rs12255372**	0.94	0.88	1	

The mean volumes of the different brain regions, according to the four analyzed SNPs and genotypes are described in Table 4. As shown in Table 5, by employing linear regression, we found a significant association in all four *TCF7L2* SNPs with amygdalar volume in the recessive genetic model (regression model B - adjusting for sex, age and total intracranial volume [TICV]): rs7901695- CC genotype vs. CT + TT genotypes, β = −0.21, p = 0.002; rs7903146-TT vs. TC + CC, β = −0.20, p = 0.003; rs11196205- CC vs. CG + GG, β = −0.247, p = 0.0003; and rs12255372- TT vs. TG + GG, β = −0.20, p = 0.003. These results withstood our threshold for multiple testing correction (p = 0.0042).

**Table 4:** Volumes (mean ± SD) of amygdala, hippocampus, gray matter and WMH according to *TCF7L2* SNPs genotypes.

*TCF7L2* SNPs [T2D risk allele]	Genotype	N	Amygdala*	Hippocampus*	Gray matter*	WMH*
rs7901695 [C]	CC	35	0.466 ± 0.082	0.496 ± 0.038	507.784 ± 60.71	13.696 ± 15.73
	CT	84	0.515 ± 0.087	0.508 ± 0.053	521.343 ± 47.95	12.542 ± 13.45
	TT	72	0.517 ± 0.079	0.508 ± 0.047	507.962 ± 47.37	13.272 ± 13.61
rs7903146 [T]	CC	77	0.515 ± 0.078	0.504 ± 0.048	508.301 ± 47.33	13.847 ± 13.88
	CT	75	0.514 ± 0.089	0.510 ± 0.052	520.995 ± 48.36	12.242 ± 13.34
	TT	35	0.466 ± 0.082	0.496 ± 0.038	507.784 ± 60.71	13.696 ± 15.73
rs11196205 [C]	CC	49	0.469 ± 0.086	0.494 ± 0.041	504.668 ± 59.42	14.490 ± 14.78
	CG	93	0.521 ± 0.080	0.512 ± 0.049	522.145 ± 45.96	12.709 ± 14.54
	GG	44	0.516 ± 0.084	0.506 ± 0.050	506.499 ± 48.84	13.038 ± 11.89
rs12255372 [T]	GG	77	0.512 ± 0.081	0.501 ± 0.052	507.759 ± 48.14	13.679 ± 13.42
	GT	82	0.518 ± 0.086	0.516 ± 0.047	523.465 ± 48.63	11.705 ± 13.43
	TT	32	0.466 ± 0.081	0.492 ± 0.038	503.657 ± 57.52	14.878 ± 16.09

*Mean of total volumes in cubic centimeters ± SD ; Abbreviations: SNP- single nucleotide polymorphism; T2D- type 2 diabetes; WMH- white matter hyperintensities.

**Table 5:** Association of *TCF7L2* SNPs with amygdala, hippocampus, gray matter and white matter hyperintensities (WMH) volume.

*TCF7L2* SNPs [T2D risk allele]	Genetic model	Regression model A				Regression model B			
		Unstandardized Coefficients		Standardized Coefficients	p value	Unstandardized Coefficients		Standardized Coefficients	p value
		B	Std. Error	β		B	Std. Error	β	
**Amygdala**									
rs7901695 [C]	Additive	−0.018	0.008	−0.155	0.0270	−0.016	0.0080	−0.140	0.0450
	Recessive	−0.046	0.015	−0.208	**0.0020**	−0.043	0.015	−0.198	**0.003**
	Dominant	−0.011	0.012	−0.060	0.395	−0.008	0.012	−0.043	0.541
rs7903146 [T]	Additive	−0.018	0.008	−0.157	0.025	−0.017	0.008	−0.147	0.036
	Recessive	−0.044	0.015	−0.202	**0.003**	−0.042	0.015	−0.192	0.005
	Dominant	−0.013	0.012	−0.073	0.307	−0.011	0.012	−0.064	0.367
rs11196205 [C]	Additive	−0.023	0.008	−0.194	0.005	−0.022	0.008	−0.181	0.008
	Recessive	−0.048	0.013	−0.247	**0.0003**	−0.044	0.013	−0.226	**0.001**
	Dominant	−0.013	0.014	−0.066	0.343	−0.013	0.014	−0.067	0.333
rs12255372 [T]	Additive	−0.016	0.008	−0.134	0.054	−0.014	0.008	−0.118	0.090
	Recessive	−0.046	0.015	−0.203	**0.003**	−0.043	0.015	−0.191	0.005
	Dominant	−0.007	0.012	−0.039	0.585	−0.004	0.012	−0.021	0.762
**Hippocampus**									
rs7901695 [C]	Additive	−0.003	0.004	−0.039	0.559	−0.002	0.004	−0.027	0.685
	Recessive	−0.010	0.008	−0.077	0.246	−0.009	0.008	−0.071	0.275
	Dominant	0.000	0.007	0.005	0.944	0.002	0.007	0.020	0.770
rs7903146 [T]	Additive	0.000	0.004	−0.002	0.978	0.000	0.004	0.004	0.950
	Recessive	−0.009	0.008	−0.069	0.300	−0.008	0.008	−0.066	0.317
	Dominant	0.005	0.007	0.055	0.425	0.006	0.007	0.061	0.363
rs11196205 [C]	Additive	−0.006	0.005	−0.085	0.205	−0.005	0.004	−0.069	0.287
	Recessive	−0.015	0.007	−0.141	0.035	−0.013	0.007	−0.121	0.064
	Dominant	0.001	0.008	0.005	0.938	0.001	0.007	0.010	0.885
rs12255372 [T]	Additive	0.001	0.005	0.015	0.822	0.002	0.004	0.032	0.628
	Recessive	−0.015	0.008	−0.116	0.079	−0.013	0.008	−0.101	0.121
	Dominant	0.011	0.007	0.115	0.090	0.013	0.007	0.130	0.052
**Gray Matter**									
rs7901695 [C]	Additive	−2.602	2.974	−0.037	0.383	−2.509	2.955	−0.036	0.397
	Recessive	−7.397	5.451	−0.057	0.176	−7.223	5.366	−0.056	0.180
	Dominant	−0.937	4.497	−0.009	0.835	−0.759	4.481	−0.007	0.866
rs7903146 [T]	Additive	−1.939	2.902	−0.028	0.505	−1.997	2.884	−0.029	0.490
	Recessive	−6.473	5.419	−0.050	0.234	−6.448	5.364	−0.050	0.231
	Dominant	−0.216	4.413	−0.002	0.961	−0.333	4.388	−0.003	0.940
rs11196205 [C]	Additive	−4.048	3.034	−0.056	0.184	−3.580	2.987	−0.050	0.232
	Recessive	−9.779	4.830	−0.085	0.044	−8.626	4.767	−0.075	0.072
	Dominant	−0.667	5.099	−0.006	0.896	−0.640	5.019	−0.005	0.899
rs12255372 [T]	Additive	−2.323	2.987	−0.033	0.438	−2.085	2.969	−0.030	0.483
	Recessive	−9.199	5.640	−0.068	0.105	−8.193	5.564	−0.061	0.143
	Dominant	0.500	4.415	0.005	0.910	0.495	4.400	0.005	0.911
**WMH**									
rs7901695 [C]	Additive	−0.149	1.428	−0.008	0.917	−0.042	1.441	−0.002	0.977
	Recessive	0.558	2.635	0.016	0.833	0.759	2.633	0.021	0.773
	Dominant	−0.709	2.147	−0.025	0.742	−0.611	2.171	−0.021	0.779
rs7903146 [T]	Additive	−1.130	1.131	−0.075	0.319	−1.131	1.130	−0.075	0.318
	Recessive	−1.822	2.139	−0.063	0.395	−1.755	2.128	−0.061	0.411
	Dominant	−1.394	1.699	−0.062	0.413	−1.423	1.692	−0.063	0.401
rs11196205 [C]	Additive	0.220	1.171	0.014	0.851	0.238	1.168	0.015	0.839
	Recessive	0.497	1.876	0.020	0.791	0.355	1.880	0.014	0.851
	Dominant	0.072	1.949	0.003	0.970	0.279	1.940	0.011	0.886
rs12255372 [T]	Additive	−0.667	1.150	−0.043	0.563	−0.711	1.157	−0.046	0.540
	Recessive	−0.511	2.208	−0.017	0.817	−0.548	2.202	−0.018	0.804
	Dominant	−1.118	1.673	−0.050	0.505	−1.184	1.682	−0.053	0.483

Each SNP was analyzed for association with the four phenotypes under additive, recessive and dominant genetic models, using linear regression. The basic regression model (A) included adjusting for age, sex and TICV (except of WMH). In addition to the covariates included in model A, the second model (B), was also adjusted for T2D related characteristics, BMI and ancestry. Significant associations are in bold. Abbreviations: SNP- single nucleotide polymorphism; T2D- type 2 diabetes; WMH- white matter hyperintensities.

Controlling also for T2D-related covariates (time in the diabetes registry, mean hemoglobin A1c (HbA1C) levels and use of T2D medication [yes/no]), body mass index (BMI) and ancestry (regression model B) did not change the results substantively (rs7901695 β = −0.2, p = 0.003; rs7903146 β = −0.19, p = 0.005; rs11196205 β = −0.23, p = 0.001; and rs12255372 β = −0.19, p = 0.005), although rs12255372 and rs7903146 no longer withstood the threshold for multiple testing correction (Table 5). In all four SNPs, individuals who were homozygous of the T2D risk allele had ~9.5% smaller amygdalar volume compared to the carriers of the non-risk allele (Table 6). Adjusting also to systolic and diastolic blood pressure values did not change results (regression model C, Supplementary Table 1). In addition, *TCF7L2* rs11196205 showed a significant association with amygdalar volume in the additive genetic model (regression model B- p = 0.005; model B- p = 0.008) but did not remain significant when implementing multiple testing correction (Table 5).

**Table 6:** Amygdalar volumes (mean ± SD) according to *TCF7L2* SNPs genotypes groups (recessive genetic model).

*TCF7L2* SNPs [T2D risk allele]	Genotype group	N	Amygdala*
rs7901695 [C]	CC	35	0.466 ± 0.082
	CT + TT	156	0.516 ± 0.083
	Volume difference		9.7%
rs7903146 [T]	CC + CT	152	0.514 ± 0.083
	TT	35	0.466 ± 0.082
	Volume difference		9.3%
rs11196205 [C]	CC	49	0.469 ± 0.086
	CG + GG	137	0.519 ± 0.081
	Volume difference		9.6%
rs12255372 [T]	GG + GT	159	0.515 ± 0.083
	TT	32	0.466 ± 0.081
	Volume difference		9.5%

*Mean of total volumes in cubic centimeters ± SD ; Abbreviations: SNP- single nucleotide polymorphism; T2D- type 2 diabetes.

In order to analyze the potential distinct effect of the four *TCF7L2* SNPs (under recessive model), we performed a conditional analysis, including a second SNP as a covariate in the regression model. In the joint analysis of rs11196205 and each of the other three SNP (separately), the association with amygdalar volume was still significant (regression model A- p = 0.039, p = 0.044, p = 0.033 – controlling for rs7901695, rs7903146 or rs12255372 respectively), or approaching significance (model B- p = 0.08, p = 0.086, p = 0.067 – controlling for rs7901695, rs7903146 or rs12255372 respectively) (Table 7). However, in the joint analysis of the highly correlated SNP rs7901695/rs7903146 with rs11196205 or rs12255372, the association of rs7901695 (model A, p = 0.61 and p = 0.3, respectively) or rs7903146 (model A, p = 0.624 and p = 0.322, respectively) with amygdalar volume became non- significant. Similarly, the joint analysis of rs12255372 with any of the other SNPs (Table 7) was not significant. Therefore, we assume that none of the three SNPs rs7901695, rs7903146 and rs12255372 contributed independently to the association with amygdalar volume, beyond the effect of the most highly significant SNP rs11196205.

**Table 7:** Conditional analysis for the association of *TCF7L2* SNPs with amygdalar volume under recessive model.

Amygdala			
SNP	Adjusted for SNP	Regression model A- p value	Regression model B- p value
rs7901695*	rs11196205	0.609	0.501
	rs12255372	0.304	0.297
rs7903146*	rs11196205	0.624	0.529
	rs12255372	0.322	0.321
rs11196205	rs7901695	**0.039**	0.08
	rrs7903146	**0.044**	0.086
	rs12255372	**0.033**	0.067
rs12255372	rs7901695	0.435	0.495
	rrs7903146	0.452	0.505
	rs11196205	0.762	0.651

The basic regression model (A) included adjusting for age, sex and TICV. In addition to the covariates included in model A, the second model (B), was also adjusted for T2D related characteristics, BMI and ancestry. Significant associations are in bold. *Conditional analysis for the rs7903146 and rs7901695 SNP pair was not performed due to the high correlation between them (r^2^ = 0.94). Abbreviations: SNP- single nucleotide polymorphism; T2D- type 2 diabetes;

Due to a variable levels of linkage disequilibrium (LD) between the four *TCF7L2* SNPs, we performed haplotype analysis. Two haplotype blocks were found: 1. rs7901695 and rs7903146 (first block); 2. rs11196205 and rs12255372 (second block). Consistent with the recessive model, participants with two copies of the first block CT haplotype (rs7901695-C and rs7903146-T, N = 32) or participant with two copies of the second block CT haplotype (rs11196205-C and rs12255372-T, N = 35) had significantly smaller amygdalar volume compared to participants with other haplotype combinations in the same block (Supplementary Table 2). These results were essentially identical to the association of rs7903146 and rs12255372 alone, respectively (model A- p = 0.003; model B- p = 0.005). Combining all SNPs, carriers of two copies of the CTCT haplotype (rs7901695-C, rs7903146-T, rs11196205-C and rs12255372-T, N = 29) had significantly smaller amygdalar volume compared to participants with other haplotypes combinations (model A- p = 0.013; model B- p = 0.014). Information regarding the haplotype analysis, including haplotypes frequencies, is presented in Supplementary Tables 2 and 3.

A posteriori power estimates (based on the observed regression coefficients) for rs11196205 association with amygdalar volume in our sample (recessive model, minor allele frequency of 0.487, required p = 0.05), ranged from 94% (adjusting for only age, sex and TICV) to 89% (adjusting also for additional covariates). A posteriori power estimates for the other three SNPs (rs7901695, rs7903146 and rs12255372) association with amygdalar volume (recessive model, minor allele frequency of 0.382–0.403, required p = 0.05), were 77–83% (model A) and 68–78% (model B).

Interestingly, we also found a significant association of rs11196205 with hippocampal and gray matter volume under the recessive genetic model at a nominal significance level (β = −0.141, p = 0.035 and β = −0.085, p = 0.044, respectively), but these results became marginal when the second set of covariates was added (model B- β = −0.121, p = 0.064 and β = −0.075, p = 0.072, respectively) (Table 5). All other associations (amygdalar volume in the additive and dominant models, as well as all models for WMH, hippocampal and gray matter volumes) did not reach the required level of significance after correction for multiple testing.

### Association of *TCF7L2* SNPs with left versus right amygdalar volume

In a secondary analysis we applied similar linear regression for left and right amygdala separately, in accordance to previous evidence in the literature showing differences between the two sides^34^. As shown in Table 8, association results of all four SNPS are stronger (at the significance level achieved) for the left amygdala under recessive model (Model B- rs7901695 β = −0.19, p = 0.005; rs7903146 β = −0.19, p = 0.006; rs11196205 β = −0.24, p = 0.0004; and rs12255372 β = −0.21, p = 0.002), while weaker for the right amygdala (Model B- rs7901695 β = −0.18, p = 0.012; rs7903146 β = −0.17, p = 0.018; rs11196205 β = −0.19, p = 0.006; and rs12255372 β = −0.16, p = 0.025). Looking at the left amygdala separately, results of conditional (Table 9) and haplotype (Supplementary Table 2) analyses are similar to that of the total amygdalar volume.

**Table 8:** Association of TCF7L2 SNPs with left and right amygdalar volume (recessive genetic model).

*TCF7L2* SNPs [T2D risk allele]	Regression model A				Regression model B			
	Unstandardized Coefficients		Standardized Coefficients		Unstandardized Coefficients		Standardized Coefficients	
	B	Std. Error	β	p value	B	Std. Error	β	p value
**Amygdala- Left**								
rs7901695 [C]	−0.053	0.0180	−0.199	**0.003**	−0.050	0.018	−0.188	0.005
rs7903146 [T]	−0.052	0.018	−0.196	**0.004**	−0.049	0.018	−0.185	0.006
rs11196205 [C]	−0.061	0.016	−0.257	**0.0001**	−0.056	0.016	−0.236	**0.0004**
rs12255372 [T]	−0.062	0.018	−0.224	**0.001**	−0.059	0.018	−0.212	**0.002**
**Amygdala- Right**								
rs7901695 [C]	−0.047	0.0180	−0.183	0.0090	−0.045	0.0180	−0.176	0.0120
rs7903146 [T]	−0.044	0.018	−0.175	0.013	−0.042	0.018	−0.168	0.018
rs11196205 [C]	−0.047	0.016	−0.211	**0.003**	−0.043	0.016	−0.192	0.006
rs12255372 [T]	−0.044	0.018	−0.167	0.017	−0.042	0.018	−0.158	0.025

The basic linear regression model (A) included adjusting for age, sex and TICV. In addition to the covariates included in model A, the second model (B), was also adjusted for T2D related characteristics, BMI and ancestry. Significant associations are in bold. Abbreviations: SNP- single nucleotide polymorphism; T2D- type 2 diabetes.

**Table 9:** Conditional analysis for the association of *TCF7L2* SNPs with left amygdalar volume under recessive model.

Left amygdala			
SNP	Adjusted for SNP	Regression model A- p value	Regression model B- p value
rs7901695*	rs11196205	0.943	0.828
	rs12255372	0.754	0.752
rs7903146*	rs11196205	0.952	0.86
	rs12255372	0.775	0.784
rs11196205	rs7901695	**0.012**	**0.027**
	rrs7903146	**0.013**	**0.027**
	rs12255372	**0.038**	0.076
rs12255372	rs7901695	0.119	0.139
	rrs7903146	0.121	0.14
	rs11196205	0.588	0.488

The basic regression model (A) included adjusting for age, sex and TICV. In addition to the covariates included in model A, the second model (B), was also adjusted for T2D related characteristics, BMI and ancestry. Significant associations are in bold. *Conditional analysis for the rs7903146 and rs7901695 SNP pair was not performed due to the high correlation between them (r^2^ = 0.94). Abbreviations: SNP- single nucleotide polymorphism; T2D- type 2 diabetes.

## Discussion

The well-established association of *TCF7L2* with T2D and the link between T2D and brain imaging changes, have motivated us study the association of this gene with neuroimaging phenotypes in our sample of elderly T2D Jewish patients. We have found a consistent association of *TCF7L2* SNPs with amygdalar volume. In the four investigated SNPs (rs7901695, rs7903146, rs11196205, and rs12255372), carriers of two copies of the T2D risk allele had smaller amygdalar volume, compared to carriers of the non-risk allele (recessive model), while controlling for sex, age and TICV. Adjusting also for T2D related covariates, BMI, ancestry and blood pressure did not change the results substantially. Further examination of the left and right amygdala separately, revealed that the association is derived mainly due to the left amygdalar volume (p = 0.0004–0.006) than the right amygdalar volume (p = 0.006–0.025). On conditional analysis, we found that rs7901695, rs12255372 or rs7903146 SNPs associations with amygdalar volume were not independent of the most highly significant SNP rs11196205, and therefore only one association signal was detected in region. No associations of hippocampal, gray matter and WMH volumes with *TCF7L2 SNPs* withstood Bonferroni adjustment for multiple testing correction.

Several limitations of this study should be considered. Our sample size (N = 191 individuals) is considered small in the context of a genetic association study. Nevertheless, the sample is unique, since it includes only T2D elderly, a population at risk for cognitive decline and dementia. All participants had clinical (including measures of glycemic control), neuroimaging and genetic data. Some of the associations survived Bonferroni correction for multiple  testing, indicating robustness of the results and therefore reducing the likelihood of false positive results. The mere nominal level of association with gray matter and hippocampal volume, which did not withstand the Bonferroni correction, might be due to a small sample size, and larger size would have been an advantage in terms of statistical power. In addition, the cross-sectional design of the study impedes reaching conclusions of causality. The longitudinal component of the IDCD is ongoing and may assist in shedding light on functional effect of this association in the future.

Previous neuroimaging genetics studies did not find association of *TCF7L2* with amygdalar volume. Of particular interest is a recent GWAS meta-analysis study of ~30,000 participants (mostly European origin) conducted by the Enhancing Neuro Imaging Genetics through Meta-Analysis (ENIGMA) consortium^35^ (which did not include specific cohorts of T2D patients in particular). No significant associations (additive model, p < 0.05) were found in this study between amygdalar volume and *TCF7L2* SNPs rs7901695, rs7903146 and rs12255372. Although rs11196205 was not tested directly in the ENIGMA GWAS, its proxy SNP rs10885409 (D′ = 1, r^2^ > 0.95), found by using the Broad institute SNP proxy search **(**http://archive.broadinstitute.org/mpg/snap/ldsearch.php**)**, was not significantly associated with amygdalar volume as well. Several explanations for the discrepancy in results are plausible in addition to different genetic model, including that the observed association of *TCF7L2* with amygdalar volume is specific to the Jewish population, or alternatively is specific to T2D affected individuals. Our study did not include control participants without T2D, and therefore we cannot address the generalization of this finding to non-T2D individuals. It is also possible that the association is influenced by older age and potentially not found in younger population (this sample include individuals aged 65 years and older). Taken together, at the current stage, our findings should be considered as preliminary and caution is required in their interpretation. Further studies are required in various populations to validate it.

The amygdala, one of the limbic system’s components, has been implicated in several functions - mainly emotional processing and responses (e.g. fear, anxiety, and aggression), decision-making, associative learning and memory^36^. Amygdalar aberrant function or structure is common in neurodevelopmental disorders^37^. Previous studies in various populations have reported association of variation in several genes with amygdalar volume, including *STMN1* and *SLC6A4*^38^, *CACNA1C*^39,40^ and the oxytocin receptor *OXTR*^41,42^.

Consistent with our results, previous reports have demonstrated an association between structural brain changes and T2D, e.g. lower brain volumes and greater brain atrophy in T2D patients^24–26^, including amygdala^30^. Indeed, greater amygdalar atrophy had been associated with high plasma glucose levels within the normal range^31^.

*TCF7L2* is expressed in many brain regions, including the amygdala in mice^43^ and at a relatively low level in human amygdala (Genotype Tissue expression portal, GTex, Broad Institute; https://gtexportal.org/home/gene/TCF7L2/). As part of the Wnt/beta-catenin signaling pathway, *TCF7L2* plays role in the activation of lymphoid enhancer-binding factor 1/T cell factor (LEF1/TCF) transcription factors complexes. The Wnt/beta-catenin signaling is involved in neuroplasticity, adult neurogenesis and CNS development^44–46^, as well as in amygdala-dependent learning and long-term memory formation^47^. Decreased levels of beta‐catenin were found in the amygdala of rats that showed behavioral sensitization to administration of drugs of abuse^48^. In humans, polymorphisms in *TCF7L2* were associated with schizophrenia and bipolar disorder^20–22^. At the behavioral level in animal models, *TCF7L2* deficient mice demonstrated altered anxiety like behavior and fear learning^49^, and this gene mediated cellular and behavioral response to lithium treatment in mice and zebrafish^50^. Combined, these evidences implicate a role of *TCF7L2* in brain function and behavioral phenotypes.

To conclude, our results in a sample of T2D elderly demonstrate for the first-time associations of four *TCF7L2 SNPs* with amygdalar volume. Confirmation of these results in additional cohorts is required in order to reach more definitive conclusions.

## Methods

### Sample

Participants were recruited from the Israel Diabetes and Cognitive Decline (IDCD) study, a collaboration of the Icahn School of Medicine, Mount Sinai, NY, USA, Sheba Medical Center, Israel, and the Maccabi Health Services (MHS), Israel. The IDCD study design has been previously described in detail^51^. Briefly, community-dwelling Israeli elderly individuals with T2D (≥65 years old) were recruited from the MHS diabetes registry. Criteria for enrolment into the IDCD study were: (1) having T2D (defined as any of the following- (A) HbA1c > 7.25%; (B) Glucose blood levels of 200 mg/dl on two examinations more than 3 months apart; (C) purchase of diabetic medication twice within 3 months; or (D) diagnosis of T2D (International Classification of Diseases [ICD9] code) by a general practitioner, internist, endocrinologist, ophthalmologist, or diabetes advisor, supported by a HbA1c > 6.5% or glucose > 125 mg/dl within half a year); (2) normal cognition at entry to the IDCD study; (3) being free of any neurological (e.g., Parkinson’s disease, stroke), psychiatric (e.g. schizophrenia) or other diseases (e.g., alcohol or drug abuse) that might affect cognition; (4) having an informant; (5) fluency in Hebrew; (6) living in the area of Tel Aviv. The Diabetes Registry has collected detailed laboratory, medication, and diagnoses information since 1998^52^. Based on self-report, the IDCD individuals are unrelated to each other (at least at first- and second-degree level). The HbA1c and blood pressure (systolic and diastolic) values were calculated for each participant as the means of all measurements in the diabetes registry.

### MRI acquisition

A randomly recruited sub-sample of the IDCD cohort underwent MRI scan, performed at the Diagnostic Imaging Department, Sheba Medical Center using a 3 Tesla scanner (GE, Signa HDxt, v16VO2). High-resolution (1 mm^3^) images were acquired by using a 3D inversion recovery prepared fast spoiled gradient-echo (FSPGR) T1-weighted sequence (TR/TE = 7.3/2.7 s, 20° flip angle, TI 450 ms). In addition, a T2-weighted fluid-attenuated inversion recovery (FLAIR) sequence was acquired with the following parameters: Repetition time/Echo time (TR/TE) 9500/123 ms, axial slices, slice-width/gap 3/0.4 mm, 22 cm FOV, 64 × 64 matrix, 90° flip-angle.

### MRI analysis

For volumetric analysis, the voxel based morphometry (VBM^53^) toolbox, (http://www.fil.ion.ucl.ac.uk/spm/ext/#VBMtools) implemented in Statistical Parametric Mapping (SPM8) software was used on the T1 weighted anatomical images. This procedure included automated iterative skull stripping, segmentation of the images into gray matter, white matter (WM), cerebrospinal fluid probability images, and spatial normalization of the gray matter images to a customized gray matter template in standard MNI (Montreal Neurological Institute) space. Finally, the gray matter maps were smoothed using an 8 mm Gaussian kernel. Gray matter probability maps were thresholded at 0.2 to minimize inclusion of incorrect tissue types. Total intracranial volume (TICV) was calculated by summing the segmented and thresholded images (TICV = gray matter + white matter + cerebrospinal fluid). Based on our a-priori hypothesis, we used a region of interest (ROI) approach centered on the amygdala and hippocampus, identified with the ‘Human Automated Anatomical Labelling (AAL) atlas’^54^ within the Wake Forest University PickAtlas (http://www.rad.wfubmc.edu/fmri) and extracted using the MarsBaR ROI toolbox^55^ as implemented in SPM12. All reported volumes are total regional volumes.

For WMH quantification we used the Lesion segmentation toolbox (LST) (implemented in SPM8), following previously described methods^32^. The default LST settings were used with the exception of κ (k), a value indicating the threshold for the initial lesion mask. Visual inspection of the probability maps across participants by using various k values, to maximize sensitivity while reducing false positive results, indicated that a k = 0.15 was the optimal value for our sample images. This procedure generated one binary lesion image per participant from which a total lesion volume (in milliliters) map was extracted.

### SNPs selection and genotyping

Four intronic *TCF7L2* SNPs (rs7901695, rs7903146, rs11196205, and rs12255372) were selected for this study (Table 2), based on ample evidence of their confirmed association with T2D and related traits^4,5^. These SNPs were genotyped with the Sequenom MassARRAY system, at the Washington University Human Genetics Division Genotyping Core, St. Louis, USA. Quality control measures were implemented.

### Statistical analysis

We employed hierarchical linear regression to study the association of the *TCF7L2* SNPs with amygdalar, hippocampal, gray matter and WMH volumes, under three genetic models (additive, dominant and recessive – referring to the effect of the T2D risk allele). In the basic regression model (model A), we controlled for sex, age at IDCD baseline recruitment and TICV (this covariate was included in the models for gray matter, hippocampus and amygdala). In the second step (model B), we included in the regression model all the covariates from model A, in addition to a set of T2D related characteristics (time in the MHS diabetes registry [an approximation to T2D duration^56^], mean HbA1C levels, use of T2D medication [yes/no]), mean body mass index (BMI), and ancestry (Ashkenazi vs. Non-Ashkenazi, based on self-report and land of birth data). In the third step (model C), we included in the regression model all the covariates from models A and B, in addition to mean systolic and mean diastolic blood pressure.

The analysis was conducted for each SNP and brain region separately. For each brain region, a two-sided p value of 0.0042 (0.05/12) was considered statistically significant following employment of Bonferroni correction for multiple testing (0.05/[4 SNPs included in the final analysis × 3 genetic models]). For statistical analysis, we used SPSS version 21.0 (SPSS Inc., Chicago, IL, USA). Hardy–Weinberg calculations, SNPs pairwise correlation and linkage disequilibrium (LD) values were obtained with PLINK (http://pngu.mgh.harvard.edu/purcell/plink)^57^. For the WMH, we applied square-root transformation to obtain normal distribution. Power calculation for *TCF7L2* SNPs association with amygdalar volume was carried by Quanto v1.2.4 software^58^. To assess a potential distinct contribution of the four *TCF7L2* SNPs on amygdalar volume, we performed conditional analysis for each SNP (adjusting for a second SNPs within the regression model, coded recessively).

For haplotype analysis, we used PLINK to determine haplotypes blocks and frequencies. We employed hierarchical linear regression to study the association of the *TCF7L2* haplotypes with amygdalar volume under recessive model (comparing carriers of two copies of haplotype of interest, to carriers of all other haplotypes combinations), adjusting for covariates.

### Study approval and informed consent

All participants provided informed consent, and all experimental protocols were approved by the institutional review boards (IRBs) of all three collaborating institutions (Icahn School of Medicine, Mount Sinai, NY, USA, Sheba Medical Center, Israel, and MHS, Israel). In addition, all the methods were carried out in accordance with the relevant guidelines and regulations.

## Supplementary information

Dataset 1

## Data Availability

The datasets generated and analyzed during the current study are available from the corresponding author on reasonable request.

## Supplementary information

is avilable for this paper at 10.1038/s41598-019-48899-3.
