# Publisher Correction: *TCF7L2* polymorphisms are associated with amygdalar volume in elderly individuals with Type 2 Diabetes

**DOI:** 10.1038/s41598-020-58945-0

**Published:** 2020-02-05

**Authors:** Ithamar Ganmore, Abigail Livny, Ramit Ravona-Springer, Itzik Cooper, Anna Alkelai, Shahar Shelly, Galia Tsarfaty, Anthony Heymann, Michal Schnaider Beeri, Lior Greenbaum

**Affiliations:** 1 Department of Neurology, Sheba Medical Center, Tel Hashomer, Ramat Gan Israel; 2 The Joseph Sagol Neuroscience Center, Sheba Medical Center, Tel Hashomer, Ramat Gan Israel; 3 Department of Diagnostic Imaging, Sheba Medical Center, Tel Hashomer, Ramat Gan Israel; 4 Memory clinic, Sheba Medical Center, Tel Hashomer, Ramat Gan Israel; 5 Sackler Faculty of Medicine, Tel Aviv University, Tel Aviv, Israel; 6 Institute for Genomic Medicine, Columbia University, New York, NY USA; 7 Department of Neurology, Mayo Clinic Rochester, Minnesota, USA; 8 Maccabi Healthcare Services, Tel Aviv, Israel; 9 Department of Psychiatry, Icahn School of Medicine at Mount Sinai, New York, NY USA; 10 The Danek Gertner Institute of Human Genetics, Sheba Medical Center, Tel Hashomer, Ramat Gan Israel

Correction to: *Scientific Reports* 10.1038/s41598-019-48899-3, published online 01 November 2019

The Supplementary Information file originally published with this Article was incorrect. This has now been updated in the HTML version of the article, the PDF version was correct at time of publication.

