# Supplementary material for: TCF7L2 polymorphisms are associated with amygdalar volume in elderly individuals with Type 2 Diabetes
Source: Sci Rep. 2019 Nov 1;9:15818. doi: 10.1038/s41598-019-48899-3 (PMC6825182; doi:10.1038/s41598-019-48899-3)
Supplement: Supplementary file 1 — Dataset 1 [file 41598_2019_48899_MOESM1_ESM.docx]

**Supplementary tables**

***TCF7L2* polymorphisms are associated with amygdalar volume in elderly individuals with Type 2 Diabetes**

Ithamar Ganmore, Abigail Livny, Ramit Ravona-Springer, Itzik Cooper, Anna Alkelai, Shahar Shelly, Galia Tsarfaty, Anthony Heymann, Michal Schnaider Beeri Lior Greenbaum^i^

**Supplementary table 1**: Model C of the association of *TCF7L2* SNPs with total amygdala, Left amygdalar and right amygdalar volumes.

| ***CF7L2* SNPs [T2D risk allele]** |  | **Regression model C** | | | | | | |
| --- | --- | --- | --- | --- | --- | --- | --- | --- |
|  | **Genetic model** | **Unstandardized Coefficients** | | | **Standardized Coefficients** | |  | |
|  |  | **B** | **Std. Error** | **Beta** | | **p value** | |  |
| **Amygdala** | | | | | | | |  |
|  | Additive | -0.017 | 0.008 | -0.142 | | 0.044 | |  |
| **rs7901695 [C]** | Recessive | -0.045 | 0.015 | -0.205 | | **0.003** | |  |
|  | Dominant | -0.008 | 0.012 | -0.043 | | 0.547 | |  |
|  |  |  |  |  | |  | |  |
|  | Additive | -0.017 | 0.008 | -.148 | | 0.036 | |  |
| **rs7903146 [T]** | Recessive | -0.043 | 0.015 | -0.199 | | **0.004** | |  |
|  | Dominant | -0.011 | 0.012 | -0.063 | | 0.376 | |  |
|  |  |  |  |  | |  | |  |
|  | Additive | -0.022 | 0.008 | -0.185 | | **0.007** | |  |
| **rs11196205 [C]** | Recessive | -0.044 | 0.013 | -0.230 | | **0.0008** | |  |
|  | Dominant | -0.014 | 0.014 | -0.071 | | 0.311 | |  |
|  |  |  |  |  | |  | |  |
|  | Additive | -0.014 | .008 | -.121 | | 0.084 | |  |
| **rs12255372 [T]** | Recessive | -0.045 | .015 | -.199 | | **0.004** | |  |
|  | Dominant | -0.004 | .012 | -.022 | | 0.753 | |  |
| **Amygdala – Left- Recessive** | | | | | | | | |
| **rs7901695 [C]** | Recessive | -0.052 | 0.018 | -0.195 | | **0.004** | |  |
| **rs7903146 [T]** | Recessive | -0.051 | 0.018 | -0.193 | | **0.005** | |  |
| **rs11196205 [C]** | Recessive | -0.057 | 0.016 | -0.240 | | **0.0004** | |  |
| **rs12255372 [T]** | Recessive | -0.060 | 0.018 | -0.217 | | **0.001** | |  |
| **Amygdala – Right- Recessive** | | | | | | | |  |
| **rs7901695 [C]** | Recessive | -0.046 | 0.018 | -0.179 | | **0.012** | |  |
| **rs7903146 [T]** | Recessive | -0.043 | 0.018 | -0.170 | | 0.018 | |  |
| **rs11196205 [C]** | Recessive | -0.044 | 0.016 | -0.195 | | **0.006** | |  |
| **rs12255372 [T]** | Recessive | -0.043 | 0.019 | -0.164 | | **0.021** | |  |

Model C is adjusted to all co-variates included in regression models A (age, sex and TICV) and B (T2D related characteristics, BMI and ancestry), in addition to mean systolic blood pressure and mean diastolic blood pressure. Significant associations are in bold.

**Supplementary table 2: TCF7L2 haplotypes analysis**

| ***TCF7L2* haplotype [T2D risk allele]** |  | **Regression model A** | | | | | **Regression model B** | | | | |  |
| --- | --- | --- | --- | --- | --- | --- | --- | --- | --- | --- | --- | --- |
|  | **Number of carriers of two copies of risk haplotype/ all other haplotype combinations (N, %)** | **Unstandardized Coefficients** | | **Standardized Coefficients** |  | **Unstandardized Coefficients** | | | **Standardized Coefficients** |  |  |  |
|  |  | **B** | **Std. Error** | **Beta** | **p value** | **B** | | **Std. Error** | **Beta** | **p value** |  |  |
| **Total amygdala** | | | | | | | | | | | | |
| CC-TT (rs7901695 [CC]- rs7903146 [TT]) | 32/154 (17.2%/ 82.8%) | -0.044 | 0.015 | -0.202 | **0.003** | -0.042 | | 0.015 | -0.192 | **0.005** |  |  |
| CC-TT  (rs11196205 [CC]- rs12255372 [TT]) | 35/152 (18.7%/ 81.3%) | -0.045 | 0.015 | -0.202 | **0.003** | | -0.043 | 0.015 | -0.192 | **0.005** | |  |
| CC-TT-CC-TT (rs7901695 [CC]- rs7903146[TT]-rs11196205 [CC]- rs12255372 [TT]) | 29/ 154 (15.8%/ 84.2%) | -0.040 | 0.016 | -0.172 | **0.013** | | -0.039 | 0.016 | -0.168 | **0.014** | |  |
| **Left amygdala** | | | | | | | | | | | | |
| CC-TT (rs7901695 [CC]- rs7903146 [TT]) | 32/154 (17.2%/ 82.8%) | -0.052 | 0.018 | -0.196 | **0.004** | | -0.049 | 0.018 | -0.185 | **0.006** | |  |
| CC-TT  (rs11196205 [CC]- rs12255372 [TT]) | 35/152 (18.7%/ 81.3%) | -0.061 | 0.019 | -0.22 | **0.001** | | -0.058 | 0.018 | -0.21 | **0.002** | |  |
| CC-TT-CC-TT (rs7901695 [CC]- rs7903146[TT]-rs11196205 [CC]- rs12255372 [TT]) | 29/ 154 (15.8%/ 84.2%) | -0.050 | 0.019 | -0.175 | **0.011** | | -0.048 | 0.019 | -0.17 | **0.012** | |  |

Linear regression for association of two copies of the haplotypes under recessive model with total and left amygdalar volumes, compared to carriers of other haplotypes combinations. Significant associations are in bold.

| ***TCF7L2* SNPs** | **Haplotype combinations** | **frequency** |
| --- | --- | --- |
|  |  |  |
| rs11196205 - rs12255372 | GG | 0.487 |
|  | CT | 0.39 |
|  | CG | 0.124 |
|  |  |  |
| rs7901695- rs7903146 | TC | 0.596 |
|  | CT | 0.388 |
|  | CC | 0.016 |
|  |  |  |
| rs7901695- rs7903146-rs11196205- rs12255372 | TCGG | 0.486 |
|  | CTCT | 0.357 |
|  | TCCG | 0.095 |
|  | TCCT, CTCG, CCCT | <0.05 |
|  |  |  |

**Supplementary table 3: Haplotypes frequencies**
